# FIRE-9 – PORT / AIO-KRK-0418: a prospective, randomized, open, multicenter Phase III trial to investigate the efficacy of adjuvant/additive chemotherapy in patients with definitely-treated metastatic colorectal cancer

**DOI:** 10.1186/s12885-022-09422-6

**Published:** 2022-04-02

**Authors:** Nathanael Raschzok, Sebastian Stintzing, Volker Heinemann, Geraldine Rauch, Jens Ricke, Matthias Guckenberger, Annika Kurreck, Annabel H. S. Alig, Arndt Stahler, Lars Bullinger, Moritz Schmelzle, Wenzel Schöning, Georg Lurje, Felix Krenzien, Oliver Haase, Beate Rau, Bernhard Gebauer, Igor M. Sauer, Johann Pratschke, Dominik P. Modest

**Affiliations:** 1 Department of Surgery, Charité – Universitätsmedizin Berlin, corporate member of Freie Universität Berlin and Humboldt-Universität Zu Berlin, Campus Charité Mitte | Campus Virchow-Klinikum, Berlin, Germany; 2 Berlin Institute of Health at Charité – Universitätsmedizin Berlin, BIH Academy, Clinician Scientist Program, Berlin, Germany; 3 Department of Hematoogy, Oncology, and Cancer Immunology (CCM/CVK), Charité – Universitätsmedizin Berlin, corporate member of Freie Universität Berlin and Humboldt-Universität Zu Berlin, Berlin, Germany; 4 German Cancer Consortium, German Cancer Research Center, Heidelberg, Germany; 5 Department of Medical Oncology, Munich & Comprehensive Cancer Center, LMU University Hospital, Munich, Germany; 6 Institute of Biometry and Clinical Epidemiology, Charité – Universitätsmedizin Berlin, corporate member of Freie Universität Berlin and Humboldt-Universität Zu Berlin, Berlin, Germany; 7 Department of Radiology, LMU University Hospital, Munich, Germany; 8 Department for Radiation Oncology, University Hospital Zurich, University of Zurich, Zürich, Switzerland; 9 Department of Radiology, Charité – Universitätsmedizin Berlin, corporate member of Freie Universität Berlin and Humboldt-Universität Zu Berlin, Berlin, Germany

**Keywords:** Colorectal cancer, Colorectal metastases, Adjuvant chemotherapy

## Abstract

**Background:**

Additive/adjuvant chemotherapy as concept after local treatment of colorectal metastases has not been proven to be successful by phase III trials. Accordingly, a standard of care to improve relapse rates and long-term survival is not established and adjuvant chemotherapy cannot be recommended as a standard therapy due to limited evidence in literature. The PORT trial aims to generate evidence that post-resection/ablation/radiation chemotherapy improves the survival in patients with metastatic colorectal cancer.

**Methods:**

Patients to be included into this trial must have synchronous or metachronous metastases of colorectal cancer—either resected (R0 or R1) and/or effectively treated by ablation or radiation within 3–10 weeks before randomization—and have the primary tumor resected, without radiographic evidence of active metastatic disease at study entry. The primary endpoint of the trial is progression-free survival after 24 months, secondary endpoints include overall survival, safety, quality of life, treatments (including efficacy) beyond study participation, translational endpoints, and others. One arm of the study comprising 2/3 of the population will be treated for 6 months with modified FOLFOXIRI or modified FOLFOX6 (investigator´s choice, depending on the performance status of the patients but determined before randomization), while the other arm (1/3 of the population) will be observed and undergo scheduled follow-up computed tomography scans according to the interventional arm.

**Discussion:**

Optimal oncological management after removal of colorectal metastases is unclear. The PORT trial aims to generate evidence that additive/adjuvant chemotherapy after definitive treatment of colorectal metastases improves progression free and overall survival in patients with colorectal cancer.

**Trial registration:**

This study is registered with clinicaltrials.gov (NCT05008809) and EudraCT (2020–006,144-18).

## Background

Application of additive or adjuvant treatment following local treatment of metastases in patients with metastatic colorectal cancer (mCRC) is real practice in oncology, but data from large phase III trials are limited. Colorectal cancer has a 5-year prevalence of 87–107/100.000 persons in Germany [1, 2]. Approximately 40–50% of all patients with colorectal cancer develop metastases and benefit from local treatment of metastases, although relapse occurs in 70–80% of these patients [3–7]. A reduction of the relapse rates would accordingly improve the long-term outcome of these patients. Local treatment (surgery, ablation, or radiation) with intent to remove one or several metastases has been integrated into the treatment algorithm of metastatic colorectal cancer [7, 8]. The evolution of these strategies and consecutive recommendations in current national and international guidelines focus on therapy of liver metastases, which are present in 75–80% of all patients with colorectal metastases. Nevertheless, other metastatic lesions are also treated accordingly if the course of disease allows for it (i.e., lung, peritoneum, bone, etc.) [9–11].

Studies investigating peri- or postoperative therapy in the context of surgery of colorectal metastases have addressed largely homogeneous cohorts of metastatic disease limited to the liver [5, 6, 12, 13]. Unfortunately, these studies have failed to define a standard of care for adjuvant chemotherapy of locally treated colorectal metastases. Several aspects have limited the implementation of systemic therapies in this regard: A pooled analysis of a EORTC phase III trial of adjuvant systemic chemotherapy after surgical resection of colorectal cancer metastases showed only a marginal statistical significance in favor of adjuvant chemotherapy with fluoropyrmidine application (bolus 5-fluorouracil) due to the reduced size of the respective trials (278 patients included, assigned to chemotherapy [*n* = 138] or surgery alone [*n* = 140]; hazard ratio = 1.32; 95% CI, 1.00 to 1.76; *p* = 0.058). Moreover, this regimen is no longer used and has been replaced by more effective and less toxic regimens (oral capecitabine or infusional 5-fluorouracil) [12]. The second major trial in mCRC investigated pre- and postoperative therapy with a combination regimen of 5-FU, folinic acid and oxaliplatin (FOLFOX) vs. surgery alone [5, 6]. Importantly, the median number of metastases resected in this trial was “1”—clearly comprising a cohort with favorable risk which due to evolution of therapy is not directly comparable to patients with resected metastases in the context of current treatment algorithms. The study described a trend towards improvement in outcome that did not translate into a clear benefit in survival. Recently, a Japanese phase II or III trial suggested that additive FOLFOX after surgical treatment of colorectal liver metastases improves disease-free survival with an unclear effect on overall survival [14]. However, similar to the EORTC-initiated trial of perioperative chemotherapy [5, 6], the majority of patients in the JCOG0603 trial presented with rather low risk characteristics based on number and size of lesions, metachronous/synchronous disease and other characteristics. Therefore, these data cannot be generalized with respect to current treatment interventions for colorectal metastases, and the implications for the proposed trial seem to be limited. Accordingly, standard of care treatment to improve the relapse rates of definitively treated colorectal metastases is not established and the current national and international guidelines for colorectal cancer do not recommend additive/adjuvant chemotherapy due to insufficient evidence on its benefit [7, 8]. The FIRE-9 – PORT / AIO-KRK-0418 trial aims to generate evidence that post-resection/ablation/radiation chemotherapy improves the survival in patients with mCRC.

## Methods/design

### Aim of the study

The main objective of this randomized multicenter trial is to generate evidence that additive/adjuvant therapy after resection, ablation or radiation of metastases with modified FOLFOXIRI or modified FOLFOX6 may improve progression-free survival (PFS) and overall survival (OS) in patients with colorectal cancer. This is of specific importance since improvements in localized but also systemic therapies have resulted in increasing numbers of patients with mCRC undergoing definitive local treatment of metastases [4, 15–20]. To support the purely clinical information, a supporting translational study will help to identify subgroups of patients that might benefit from systemic therapy after definitive treatment of colorectal metastases.

### Sample size and follow-up

The primary endpoint will be PFS (progression-free survival, defined as progression/relapse or death from any cause) at 24 months after randomization.

Our sample size planning is based on two pooled, early-stopped trials in the adjuvant/additive setting using fluoropyrimidine monotherapies [12] that reported a hazard ratio for PFS in favor of active treatment of 0.76 (the originally reported hazard ratio was reported reversed as 1.32) that translated into a similar effect for the endpoint overall survival. We hypothesize a slightly larger effect for PFS in favor of active therapy due to the 2–3 drug regimens resulting in an estimated hazard ration of 0.70.

For the control arm (surgery alone) with structured follow-up, a progression/relapse/death-free rate of 40% at time point 24 months was observed translating into a 60% progression/relapse/death-rate at this time (according to the reported control arm [12]. With a hazard ratio 0.70 (= λ_I_/λ_C_ C = 0.0267/0.0382) favoring active treatment, the hypothesized relapse rate at 24 months in the intervention arm is assumed to be 47%. With a power of 80%, a 2-sided alpha of 0.05, a total of 276 events need to be observed in order to detect a difference in progression-free survival of a hazard ratio of 0.70 – favoring active treatment vs. observation (Schoenfeld formula). Assuming an accrual time of 48 months and a follow-up time of 24 months, a drop-out/censoring rate of 30% after 24 months after randomization, a total of 480 patients (320/160 in the respective arms, rounded to receive integers and maintain the allocation ratio) is expected to yield the required number of events if the accrual rate is constant. We account for additional 5% of patients that directly leave the study after randomization and never receive the study medication. In total, 507 patients (480/507≈0.95) are planned to be recruited.

### Selection of study population

#### Study population

The study will pragmatically recruit all patients with mCRC that were effectively treated with surgery/ablation/radiation, as the underlying question applies to all types of metastases in the setting of colorectal cancer. Besides this selection, the indicated criteria take into account that patients have to be fit enough to undergo an intervention.

#### Inclusion criteria

1. Patient’s signed informed consent.
2. Patient’s age ≥ 18 years at the time of signing the informed consent.
3. Histologically confirmed adenocarcinoma of the colon or rectum.
4. Resected (R0 or R1) and/or effectively treated metastases (all techniques allowed) of colorectal cancer within 3–10 weeks before randomization and resected primary tumor (synchronous or metachronous).
5. Absence of significant active wound healing complications (if applicable) prior to randomization. Resolved wound healing complications after resection/ablation are acceptable for inclusion into the trial.
6. No radiographic evidence of active metastatic disease at study entry in a computed tomography (CT) and/or magnetic resonance imaging (MRI) scan not older than 8 weeks. Pre-surgery/ablation images are eligible for the study if all lesions have been addressed in the interval.
7. ECOG performance status 0–2.
8. Adequate bone marrow, hepatic and renal organ function, defined by the following laboratory test results:
  a Absolute neutrophil count ≥ 1.5 × 10^9^/L (1500/μL)
  b Hemoglobin ≥ 80 g/L (8 g/dL)
  c Platelet count ≥ 100 × 10^9^/L (100,000/μL) without transfusion
  d Total serum bilirubin of ≤ 1.5 × upper limit of normal (ULN)
  e Aspartate aminotransferase (AST/GOT) ≤ 3.0 × ULN.
  f Calculated glomerular filtration rate (GFR) according to Cockcroft-Gault formula or according to MDRD ≥ 50 mL/min or serum creatinine ≤ 1.5 × ULN
9. Patients without anticoagulation need to present with an INR < 1.5 × ULN and PTT < 1.5 × ULN. Patient with prophylactic or therapeutic anticoagulation are allowed into the trial.
10. Proficient fluorouracil metabolism as defined:
  a Prior treatment with 5-FU or capecitabine without unusual toxicity or
  b If tested, normal DPD deficiency test according to the standard of the study site or
  c If tested, in patients with DPD deficiency test with a CPIC activity score of 1.0–1.5 fluoropyrimidine dosage should be reduced by 50%
11. For women of childbearing potential: negative pregnancy test within 14 days before randomization and agreement to remain abstinent (refrain from heterosexual intercourse) or use contraceptive methods with a failure rate of < 1% per year during the treatment period and for at least 6 months after the last dose of study treatment. For men: With female partners of childbearing potential, men must remain abstinent or use a condom plus an additional contraceptive method that together result in a failure rate of < 1% per year during the treatment period and for 6 months after the last dose of study treatment. Men must refrain from donating sperm during this same period.

#### Exclusion criteria

1. Treatment of metastases greater than 3 cm with radio-frequency/microwave ablation within 24 months prior to study entry if applicable.
2. Treatment of metastases greater than 5 cm with radiation (stereotactic/ brachytherapy) within 24 months prior to study entry if applicable.
3. Previous chemotherapy for metastatic or localized disease with > 6 cycles of FOLFOX (or FOLFOXIRI) or > 4 cycles of CAPOX/XELOX.
4. New York Heart Association Class III or greater heart failure by clinical judgement.
5. Myocardial infarction within 6 months prior to randomization; percutaneous transluminal coronary angioplasty with or without stenting within 6 months prior to randomization.
6. Unstable angina pectoris.
7. Unstable cardiac arrhythmia > grade 2 NCI CTCAE despite anti-arrhythmic therapy.
8. Ongoing toxicities > grade 2 NCI CTCAE, in particular peripheral neuropathy.
9. Active uncontrolled infection by investigator’s perspective.
10. Severe chronic non-healing wounds, ulcerous lesions or untreated bone fracture.
11. Known hypersensitivity to 5-FU, leucovorin, irinotecan or oxaliplatin
12. Bone marrow depression after radio- or chemotherapy.
13. Severe kidney dysfunction (creatinine clearance < 30 ml/min) or changes in blood count.
14. Recent or concomitant treatment with brivudine.
15. Peripheral sensitive neuropathy with functional impairment (> grade 1 acc. to CTCAE version 5.0)
16. Inflammatory bowel disease and/or bowel obstruction.
17. Simultaneous application of Johannis herbs preparations.
18. Pernicious or other megaloblastic anemia caused by vitamin B12 deficiency.
19. If tested, DPD deficiency test with a CPIC activity score < 1.
20. Major surgical procedure, open biopsy, or significant traumatic injury within 21 days prior to randomization, or abdominal surgery, invasive abdominal interventions or significant abdominal traumatic injury within 21 days prior to randomization or anticipation of need for major surgical procedure during the course of the study or non-recovery from side effects of any such procedure.
21. Any other disease, metabolic dysfunction, physical examination finding, or clinical laboratory finding that contraindicates the use of an investigational drug, may affect the interpretation of the results, or may render the patient at high risk from treatment complications.
22. Medical history of malignant disease other than mCRC with the following exceptions:
  patients who have been disease-free for at least three years before randomization
  patients with adequately treated and completely resected basal cell or squamous cell skin cancer, in situ cervical, breast or prostate cancer, stage I uterine cancer
  patients with any treated or untreated malignant disease that is associated with a 5-year survival prognosis of ≥ 90% and does not require active therapy
23. Known alcohol or drug abuse.
24. Pregnant or breastfeeding females.
25. Participation in a clinical trial or experimental drug treatment within 28 days prior to potential inclusion in the clinical trial or within a period of 5 half-lives of the substances administered in a clinical trial or during an experimental drug treatment prior to potential inclusion in the clinical trial, depending on which period is longest, or simultaneous participation in another clinical trial while taking part in this clinical trial.
26. Patients depending on sponsor, investigator or study site.
27. Suspected SARS-CoV-2 infection with or without symptoms (evaluation according to local policy in respective center with respect to actual status of pandemic and with reference to the policy that would apply to patients with similar therapy outside the trial). This may include assessment of vaccination status, anamnesis, physical examination and potentially antigen and/or PCR testing.
28. Patient committed to an institution by virtue of an order issued either by the judicial or the administrative authorities.
29. Limited legal capacity

#### Randomization

Screened and eligible patients will be included in the trial after initiation of the study. Patients will be allocated in a strictly concealed way by 2:1 randomization (intervention versus control) with variable block lengths randomization. Randomization will be stratified by four binary stratification variables, using secuTrial® (interactive Systems GmbH, Berlin, Germany):

- number of treated metastases (> 2 versus 1–2)
- pretreatment with systemic therapy for metastatic colorectal cancer yes versus no pretreatment
- choice of potential therapy in the trial: (fit for mFOLFOXIRI versus fit for FOLFOX)
- presence of at least one bad prognostic factor (peritoneal metastases resected/ known BRAF mutation/ synchronous metastases defined as evidence of metastases < 12 months vs 12 + months after first diagnosis) versus no bad prognostic factor)

#### Primary endpoint

Progression-free survival (PFS) is the primary endpoint of the trial. PFS is defined as time from randomization to death or evidence of disease relapse/progression, whatever occurs first within a time frame of 24 months.

#### Secondary endpoints

- PFS in patients with/without prior systemic therapy
- PFS in patients with R1 vs R0 resected lesions as well as ablated vs. purely resected lesions
- Overall survival
- Safety
- Quality of life
- Treatments (including efficacy) beyond study participation
- PFS and OS according to circulating tumor DNA at baseline (ctDNA positive vs. negative),
- Outcome in molecular subgroups,
- Local control of lesions according to ablative technique (surgery vs. ablation vs. radiation).

Blood samples are collected during study and follow-up to create a biobank of patients with and without relapse. Moreover, the relapses will be recorded as part of the study protocol, including the collection of tumor tissue and blood samples, if available, at relapse. The patterns of relapse will be correlated with the initially resected/ablated metastases clinically and in terms of tumor characteristics (mutations, expressions).

### Interventions and comparisons

#### Trial design

**Fig. 1:**
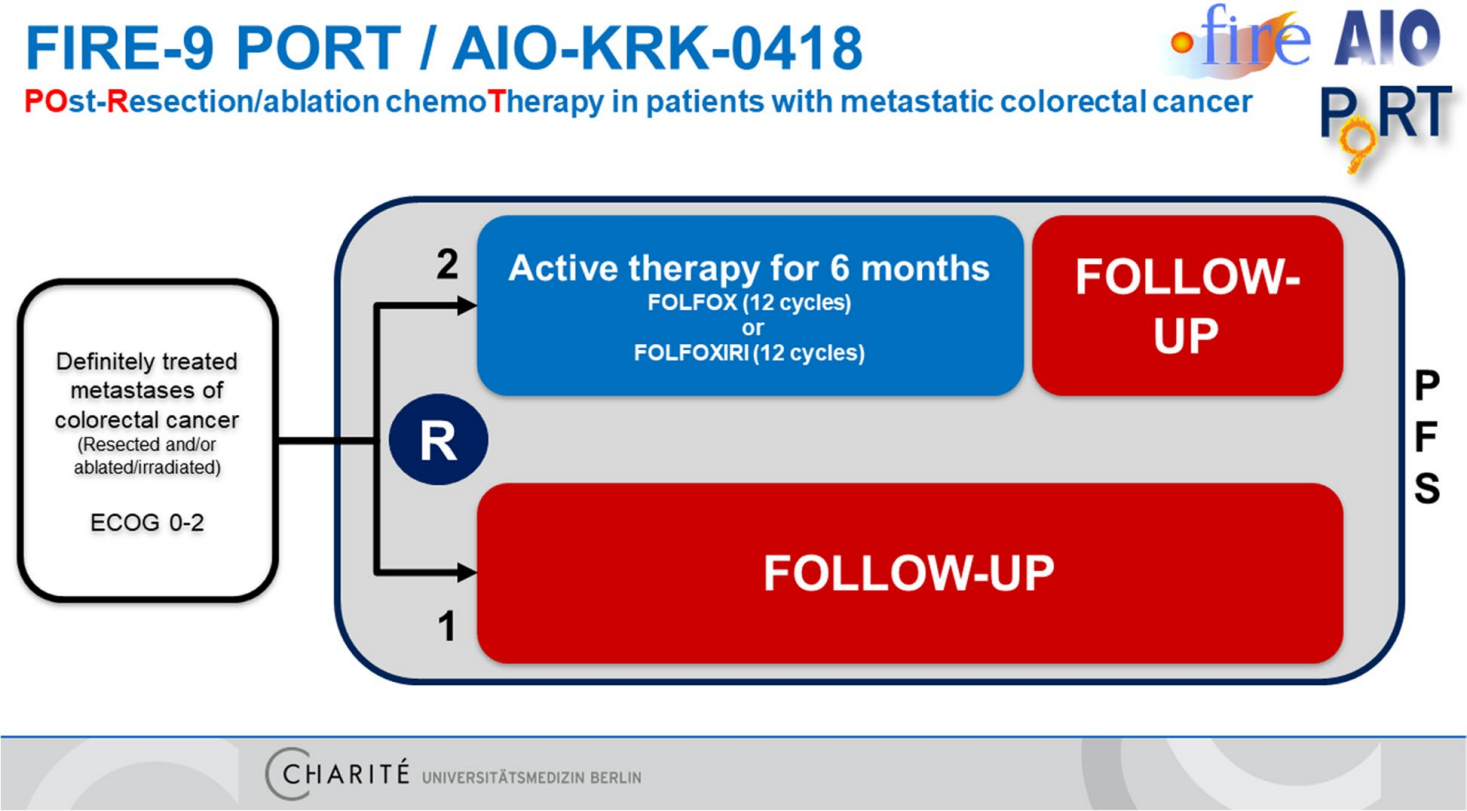
Trial design

The design of the trial is displayed in Fig. 1.

#### Experimental intervention

One arm of the study comprising 2/3 of the population will be treated (intervention) by a highly active triplet-regimen (mFOLFOXIRI) or a standard adjuvant regimen (mFOLFOX6), developed both in palliative and adjuvant treatment settings of gastrointestinal cancer [21–24]. A guidance is outlined in Table 1, however, at the discretion of the investigator, site specific modifications are permitted, e.g. additional 5-FU bolus, parallel leucovorin and oxaliplatin administration, or variation of the infusion rates. The choice of mFOLFOXIRI/mFOLFOX6 is based on the fact that available data with monotherapy have not provided a standard therapy as they failed to demonstrate superior outcome. Moreover, the success and feasibility of these regimens in adjuvant treatment of colorectal and pancreatic cancer highlights the potential of active treatment of micrometastases to prolong the overall survival. In particular—and certainly similar to pancreatic cancer—the high risk of relapse in the study cohort requires active therapy, aiming to definitely improve the outcome of patients given the risks of this treatment. The postoperative treatment period of six months was chosen based on the recommendation for adjuvant treatment in high-risk patients with UICC III colorectal cancer [25]. As the option of pre-operative therapy is left open to the discretion of the investigators, the study will limit the use of oxaliplatin to a total of 12 cycles FOLFOX(IRI) or 8 cycles of a capecitabine-based 3-weekly regimen), including the pre-study and study treatment. Accordingly, patients with more than 3 months (i.e., 6 cycles of FOLFOX[IRI] or 4 cycles of CAPOX/XELOX) pretreatment will be excluded from study participation.

**Table 1:** Trial medication (guidance for administration)

**Drug**	**Dose/** **Potency**	**Duration of administration**^c^	**Route of Administration**	**Day(s) of application**
mFOLFOX-6 regimen:				
**Oxaliplatin**	85 mg/m^2^	2 h	IV infusion	d1 of each chemotherapy cycle
**Leucovorin**	400 mg/m^2^	1-2 h	IV infusion	
**5-FU**^a^	400 mg/m^2^ bolus^b^ 2400 mg/m^2^	2–5 min 46 h	IV infusion IV infusion	
mFOLFOXIRI regimen:				
**Oxaliplatin**	85 mg/m^2^	2 h	IV infusion	d1 of each chemotherapy cycle
**Irinotecan**	150 mg/m^2^	90 min	IV infusion	
**Leucovorin**	400 mg/m^2^	1-2 h	IV infusion	
**5-FU**^a^	2400 mg/m^2^	46 h	IV infusion	

^a^in DPD mutation carriers with a CPIC activity score of 1.0–1.5, 5-FU Dosage should be reduced by 50%

^b^additional 400 mg/m^2^ bolus is permitted

^c^infusion rates of chemotherapeutical components represent recommendations but might be modified according to local standards

#### Control intervention

Since there is no established standard and no evidence of clinical improvement by systemic treatment after treatment of metastases from colorectal cancer, the control arm (1/3 of the population) will offer a structured oncological follow-up with computed tomography (CT) scans of chest and abdomen every 3 months [7].

#### Re-assessment during active study participation

Radiologic re-assessment with contrast-enhanced CT scans is scheduled 3 months (6 cycles of therapy or 3 months observation) and 6 months (12 cycles of therapy or 6 months of observation) after randomization.

#### Structured follow-up

**Fig. 2:**
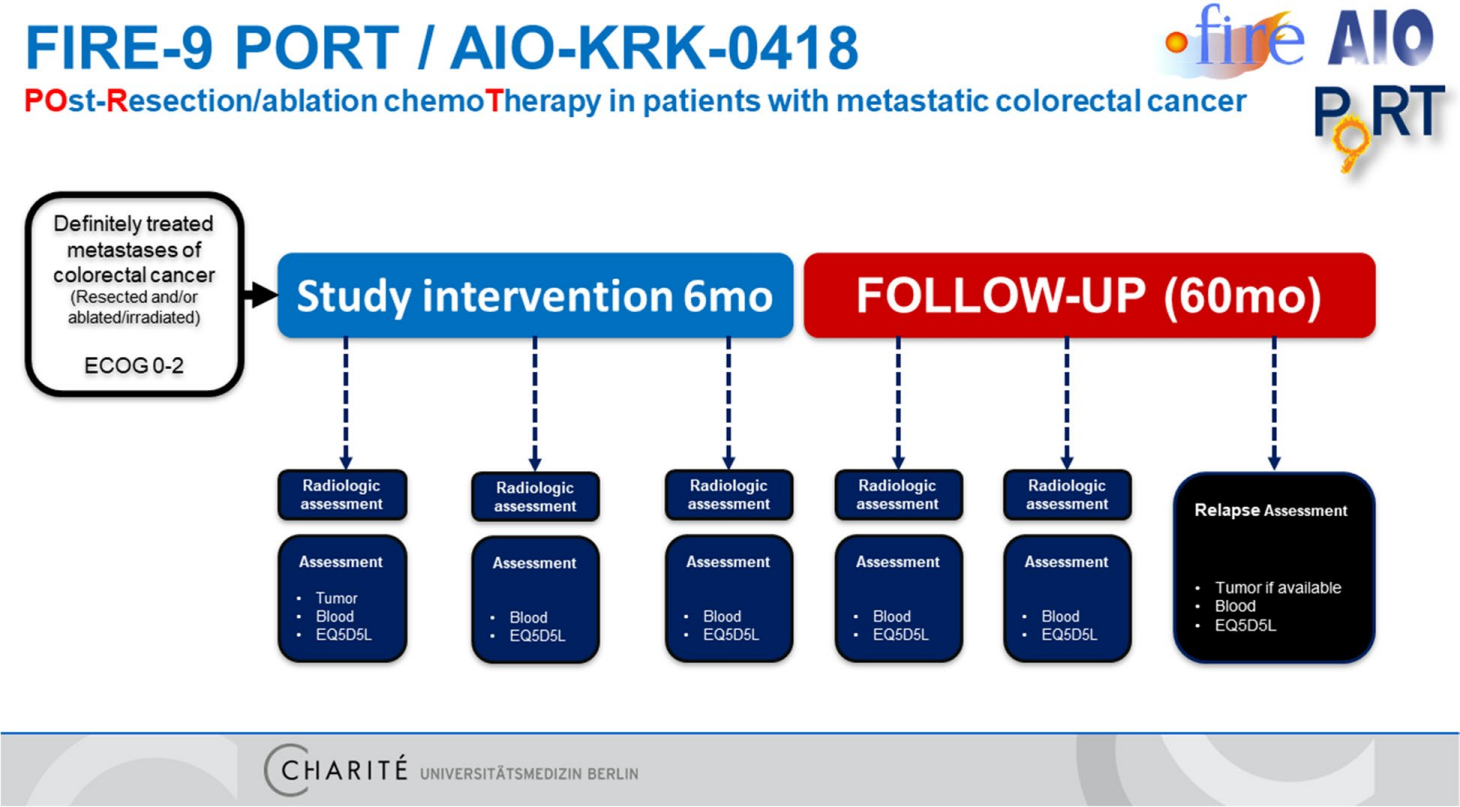
Study visits

Structured follow-up for up to 60 months after randomization should be maintained for both arms of the trial (Fig. 2). It is recommended to perform CT scans and/or MRI scans every 3 months within the 2 years after randomization. After two years without relapse, intervals are stretched to 6 months in the third and following years after randomization.

### Statistical and safety analyses

#### Primary analysis

The null hypothesis to be tested in confirmatory analysis states that the hazard ratio for PFS comparing intervention versus control equals 1. This hypothesis will be tested by means of Cox-regression adjusting for the below mentioned strata for randomization. The two-sided significance level is set to 0.05. The primary analysis will be conducted based on the full analysis set defined as the intention to treat population including all randomized patients who received the treatment at least once.

#### Secondary analyses

As a sensitivity analysis to the primary efficacy analysis, the same test will be repeated on the per-protocol population including all patients without major protocol violations. As another sensitivity analysis, the primary hypothesis will also be tested with the test for the average hazard ratio1 to potentially account for non-proportional hazards. Event probabilities of PSF will be estimated by Kaplan–Meier-Curves. The above analyses will be repeated for overall survival (OS).

#### Safety analyses

Absolute and relative frequencies as well as unbiased event-rate estimates (Kaplan–Meier, Empirical cumulative incidence) of AEs, SAEs, event rates of grade 3 and 4 toxicities (NCI-CTCAE) and abnormal biochemical paramters between treatment arms will be reported at different time points together with 95%-confidence intervals. All analyses will be done using validated statistical software.

## Discussion

Patients with locally treated metastases of colorectal cancer represent a high-risk cohort for development of recurrent metastases and consecutively death. A definite answer to the question of postoperative systemic chemotherapy is not only scientifically interesting but also an unmet clinical need with increasing prevalence. Therefore, intervention with systemic chemotherapy appears clearly justified, taking into account, that the chosen combination regimen (fluoropyrimidine plus oxaliplatin and optionally also irinotecan) represent standard of care in advanced colorectal cancer. The addition of postoperative therapy is often discussed in tumor boards and chosen for individualized treatment, in particular in previously untreated or shortly treated patients. Participating patients will receive either standard of care on the basis of available evidence, or as intervention, up to 6 months of therapy. Therefore, a potential disadvantage, although possible, seems unlikely. The PORT trial will therefore either provide a basis for routine treatment or alternatively confirm that additive/adjuvant chemotherapy after definitive treatment of colorectal metastases does not provide a clinically relevant benefit and should not be promoted.

## Data Availability

Not applicable.

## Abbreviations

CAPOX: Capecitabine plus oxaliplatin
DNA: Deoxyribonucleic acid
ECOG: Eastern cooperative oncology group
FOLFOX: Leucovorin calcium (folinic acid), fluorouracil, and oxaliplatin
FOLFOXIRI: Folinic acid, 5-fluorouracil, oxaliplatin and irinotecan
mCRC: Metastatic colorectal cancer
mFOLFOX6: 5-Fluorouracil, leucovorin, and oxaliplatin
mFOLFOXIRI: Folinic acid, 5-fluorouracil, oxaliplatin, and irinotecan
OS: Overall survival
PFS: Progression free survival
RECIST: Response evaluation criteria in solid tumours
XELOX: Capecitabine (Xeloda) and oxaliplatin

## References

[1] German Centre for Cancer Registry Data. [https://www.krebsdaten.de/Krebs/DE/Content/Krebsarten/Darmkrebs/darmkrebs_node.html;jsessionid=52586593B39EADA54D4FA96C1CBAC853.internet081]. Accessed 23 Nov 2021.

[2] Soerjomataram I, Lortet-Tieulent J, Parkin DM, Ferlay J, Mathers C, Forman D, Bray F (2012). Global burden of cancer in 2008: a systematic analysis of disability-adjusted life-years in 12 world regions. Lancet.

[3] Folprecht G, Gruenberger T, Bechstein W, Raab HR, Weitz J, Lordick F, Hartmann JT, Stoehlmacher-Williams J, Lang H, Trarbach T (2014). Survival of patients with initially unresectable colorectal liver metastases treated with FOLFOX/cetuximab or FOLFIRI/cetuximab in a multidisciplinary concept (CELIM study). Ann Oncol.

[4] Kopetz S, Chang GJ, Overman MJ, Eng C, Sargent DJ, Larson DW, Grothey A, Vauthey J-N, Nagorney DM, McWilliams RR (2009). Improved Survival in Metastatic Colorectal Cancer Is Associated With Adoption of Hepatic Resection and Improved Chemotherapy. J Clin Oncol.

[5] Nordlinger B, Sorbye H, Glimelius B, Poston GJ, Schlag PM, Rougier P, Bechstein WO, Primrose JN, Walpole ET, Finch-Jones M (2008). Perioperative chemotherapy with FOLFOX4 and surgery versus surgery alone for resectable liver metastases from colorectal cancer (EORTC Intergroup trial 40983): a randomised controlled trial. Lancet.

[6] Nordlinger B, Sorbye H, Glimelius B, Poston GJ, Schlag PM, Rougier P, Bechstein WO, Primrose JN, Walpole ET, Finch-Jones M (2013). Perioperative FOLFOX4 chemotherapy and surgery versus surgery alone for resectable liver metastases from colorectal cancer (EORTC 40983): long-term results of a randomised, controlled, phase 3 trial. Lancet Oncol.

[7] German Guideline Program in Oncology: Evidenced-based Guideline for Colorectal Cancer. Version 2.1, AWMF-Registration Number: 021/007OL; 2019.

[8] Van Cutsem E, Cervantes A, Adam R, Sobrero A, Van Krieken JH, Aderka D, Aranda Aguilar E, Bardelli A, Benson A, Bodoky G (2016). ESMO consensus guidelines for the management of patients with metastatic colorectal cancer. Ann Oncol.

[9] Carpizo DR, D’Angelica M (2009). Liver resection for metastatic colorectal cancer in the presence of extrahepatic disease. Lancet Oncol.

[10] Gonzalez M, Poncet A, Combescure C, Robert J, Ris HB, Gervaz P (2012). Risk Factors for Survival after Lung Metastasectomy in Colorectal Cancer Patients: A Systematic Review and Meta-Analysis. Ann Surg Oncol.

[11] Patel D, Townsend AR, Karapetis C, Beeke C, Padbury R, Roy A, Maddern G, Roder D, Price TJ (2016). Is Survival for Patients with Resectable Lung Metastatic Colorectal Cancer Comparable to Those with Resectable Liver Disease? Results from the South Australian Metastatic Colorectal Registry. Ann Surg Oncol.

[12] Mitry E, Fields ALA, Bleiberg H, Labianca R, Portier G, Tu D, Nitti D, Torri V, Elias D, O’Callaghan C (2008). Adjuvant Chemotherapy After Potentially Curative Resection of Metastases From Colorectal Cancer: A Pooled Analysis of Two Randomized Trials. J Clin Oncol.

[13] Primrose J, Falk S, Finch-Jones M, Valle J, O’Reilly D, Siriwardena A, Hornbuckle J, Peterson M, Rees M, Iveson T (2014). Systemic chemotherapy with or without cetuximab in patients with resectable colorectal liver metastasis: the New EPOC randomised controlled trial. Lancet Oncol.

[14] Kanemitsu Y, Shimizu Y, Mizusawa J, Inaba Y, Hamaguchi T, Shida D, Ohue M, Komori K, Shiomi A, Shiozawa M (2021). Hepatectomy Followed by mFOLFOX6 Versus Hepatectomy Alone for Liver-Only Metastatic Colorectal Cancer (JCOG0603): A Phase II or III Randomized Controlled Trial. J Clin Oncol.

[15] Choti MA, Thomas M, Wong SL, Eaddy M, Pawlik TM, Hirose K, Weiss MJ, Kish J, Green MR (2015). Surgical Resection Preferences and Perceptions among Medical Oncologists Treating Liver Metastases from Colorectal Cancer. Ann Surg Oncol.

[16] Cremolini C, Loupakis F, Antoniotti C, Lupi C, Sensi E, Lonardi S, Mezi S, Tomasello G, Ronzoni M, Zaniboni A (2015). FOLFOXIRI plus bevacizumab versus FOLFIRI plus bevacizumab as first-line treatment of patients with metastatic colorectal cancer: updated overall survival and molecular subgroup analyses of the open-label, phase 3 TRIBE study. Lancet Oncol.

[17] Cremolini C, Casagrande M, Loupakis F, Aprile G, Bergamo F, Masi G, Moretto RR, Pietrantonio F, Marmorino F, Zucchelli G (2017). Efficacy of FOLFOXIRI plus bevacizumab in liver-limited metastatic colorectal cancer: A pooled analysis of clinical studies by Gruppo Oncologico del Nord Ovest. Eur J Cancer.

[18] Heinemann V, von Weikersthal LF, Decker T, Kiani A, Vehling-Kaiser U, Al-Batran S-E, Heintges T, Lerchenmüller C, Kahl C, Seipelt G (2014). FOLFIRI plus cetuximab versus FOLFIRI plus bevacizumab as first-line treatment for patients with metastatic colorectal cancer (FIRE-3): a randomised, open-label, phase 3 trial. Lancet Oncol.

[19] Luo LX, Yu ZY, Huang JW, Wu H (2014). Selecting patients for a second hepatectomy for colorectal metastases: An systemic review and meta-analysis. Eur J Surg Oncol.

[20] Modest DP, Denecke T, Pratschke J, Ricard I, Lang H, Bemelmans M, Becker T, Rentsch M, Seehofer D, Bruns CJ (2018). Surgical treatment options following chemotherapy plus cetuximab or bevacizumab in metastatic colorectal cancer—central evaluation of FIRE-3. Eur J Cancer.

[21] André T, Boni C, Mounedji-Boudiaf L, Navarro M, Tabernero J, Hickish T, Topham C, Zaninelli M, Clingan P, Bridgewater J (2004). Oxaliplatin, Fluorouracil, and Leucovorin as Adjuvant Treatment for Colon Cancer. N Engl J Med.

[22] Conroy T, Desseigne F, Ychou M, Bouché O, Guimbaud R, Bécouarn Y, Adenis A, Raoul J-L, Gourgou-Bourgade S, de la Fouchardière C (2011). FOLFIRINOX versus Gemcitabine for Metastatic Pancreatic Cancer. N Engl J Med.

[23] Conroy T, Hammel P, Hebbar M, Ben Abdelghani M, Wei AC, Raoul J-L, Choné L, Francois E, Artru P, Biagi JJ (2018). FOLFIRINOX or Gemcitabine as Adjuvant Therapy for Pancreatic Cancer. N Engl J Med.

[24] Loupakis F, Cremolini C, Masi G, Lonardi S, Zagonel V, Salvatore L, Cortesi E, Tomasello G, Ronzoni M, Spadi R (2014). Initial Therapy with FOLFOXIRI and Bevacizumab for Metastatic Colorectal Cancer. N Engl J Med.

[25] Grothey A, Sobrero AF, Shields AF, Yoshino T, Paul J, Taieb J, Souglakos J, Shi Q, Kerr R, Labianca R (2018). Duration of Adjuvant Chemotherapy for Stage III Colon Cancer. N Engl J Med.

